# Performance of the Deep Neural Network *Ciloctunet*, Integrated with Open-Source Software for Ciliary Muscle Segmentation in Anterior Segment OCT Images, Is on Par with Experienced Examiners

**DOI:** 10.3390/diagnostics12123055

**Published:** 2022-12-06

**Authors:** Torsten Straßer, Sandra Wagner

**Affiliations:** 1 Institute for Ophthalmic Research, University of Tuebingen, 72076 Tuebingen, Germany; 2 University Eye Hospital Tuebingen, 72076 Tuebingen, Germany

**Keywords:** ciliary muscle, anterior segment, optical coherence tomography, U-Net, deep learning, image segmentation

## Abstract

Anterior segment optical coherence tomography (AS-OCT), being non-invasive and well-tolerated, is the method of choice for an in vivo investigation of ciliary muscle morphology and function. The analysis requires the segmentation of the ciliary muscle, which is, when performed manually, both time-consuming and prone to examiner bias. Here, we present a convolutional neural network trained for the automatic segmentation of the ciliary muscle in AS-OCT images. *Ciloctunet* is based on the Freiburg U-net and was trained and validated using 1244 manually segmented OCT images from two previous studies. An accuracy of 97.5% for the validation dataset was achieved. *Ciloctunet’s* performance was evaluated by replicating the findings of a third study with 180 images as the test data. The replication demonstrated that *Ciloctunet* performed on par with two experienced examiners. The intersection-over-union index (0.84) of the ciliary muscle thickness profiles between *Ciloctunet* and an experienced examiner was the same as between the two examiners. The mean absolute error between the ciliary muscle thickness profiles of *Ciloctunet* and the two examiners (35.16 µm and 45.86 µm) was comparable to the one between the examiners (34.99 µm). A statistically significant effect of the segmentation type on the derived biometric parameters was found for the ciliary muscle area but not for the selective thickness reading (“perpendicular axis”). Both the inter-rater and the intra-rater reliability of *Ciloctunet* were good to excellent. *Ciloctunet* avoids time-consuming manual segmentation, thus enabling the analysis of large numbers of images of ample study cohorts while avoiding possible examiner biases. *Ciloctunet* is available as open-source.

## 1. Introduction

Anterior segment optical coherence tomography (AS-OCT) has become the method of choice for an in vivo investigation of the ciliary muscle, mostly because it is non-invasive and well-tolerated, in contrast to alternative methods like ultrasound biomicroscopy (UBM) or magnetic resonance imaging (MRI). For a review of AS-OCT, its application, and a comparison with UBM and MRI, see [1,2]. Several groups, including ours, utilized AS-OCT to investigate different aspects of the ciliary muscle’s morphology and function, e.g., the changes in the ciliary muscle’s thickness during accommodation [3,4,5,6,7,8,9,10], the ciliary muscle’s thickness [11,12,13], movement during contraction in emmetropes and myopes [10,12,13], the relation between the ciliary muscle’s thickness and refractive error [14,15,16,17], or the ciliary muscle’s thickness and lens tension during accommodation [18]. Furthermore, the association between the axial length and ciliary muscle’s length [19], the age-related effects on the ciliary muscle’s morphology [6,16], and the impact of the prolonged nearwork on the ciliary muscle’s morphology in myopic and emmetropic eyes [20] have previously been examined. The anatomy is commonly analyzed by measuring the ciliary muscle’s thickness at a single position only [7,18,21], at equidistant steps posterior to the scleral spur [3,4,5,6,8,9,11,14,15,17,22], or proportionally to the length of the muscle [16,19,23]. Only a few studies have used narrower reading steps [8,24] or determined continuous thickness profiles [10]. Alternatively, the cross-sectional area of the ciliary muscle was assessed [22,25,26,27,28,29,30]. To facilitate the comparison of the results of different studies, suggestions have been made to harmonize the analysis of the ciliary muscle [31]. However, most methods have in common that they require either a manual placement of at least one landmark or the manual segmentation of the entire ciliary muscle within the OCT image. This is often performed using the built-in calipers of the device manufacturer’s software or image editing software, which is tedious, time-consuming, and prone to examiner bias. Custom software has been developed to ease and partly automate this task [22,23], but without being made publicly available. Our group has recently released open-source software for the semi-automated segmentation of the ciliary muscle in OCT images and the automated analysis of the biometric parameters [32], which has been employed successfully in previously published studies [10,12,20]. The software leverages manually placed guiding landmarks to find the largest brightness gradients along the ciliary muscle’s borders for fitting polynomial splines. It supports the examiner in the segmentation workflow and provides a batch processing mode to automate the extraction of the biometric parameters. However, the processing of large amounts of OCT images still requires a considerable amount of time: an experienced examiner needs about one hour to segment 10–20 images.

To eliminate the need for manual interventions for the segmentation and to avoid examiner bias, we trained a convolutional neural network based on the Freiburg U-Net’s architecture [33], using 1244 segmentations from two previous studies. The performance of the trained network *Ciloctunet* was evaluated by comparing the ciliary muscle’s biometric parameters of the OCT images of a third study segmented by the network with those resulting from segmentations originally done by two examiners. Furthermore, the results of the third study were replicated [10].

## 2. Materials and Methods

### 2.1. Training, Validation, and Test Image Data

#### 2.1.1. Imaging Protocol

The OCT images used for the training, validation, and testing of the deep neural network were taken from three previous studies: an analysis of the morphological changes in the ciliary muscle during accommodation (0D and 3D) in 15 near-emmetropic volunteers (dataset A, 180 images) [10]; a comparison of the morphological changes in the ciliary muscle of 18 emmetropic and 20 myopic volunteers for different accommodative demands (0D, 2.5D, 3D, and 4D) (dataset B, 769 images) [12]; and an investigation of the effect of a prolonged nearwork on the ciliary muscle’s morphology in 18 myopic and 17 emmetropic volunteers (0.25 D, 4 D; pre-/post-near work) (dataset C, 475 images) [20]. The participants of study C also took part in study B.

In all studies, the temporal ciliary muscle of the right eye was imaged with an anterior segment OCT (Visante AS-OCT, Carl Zeiss Meditec AG, Jena, Germany). The right eye was chosen because of the space constraints of the experimental setup. The detailed experimental setup is described in [10,12]. The acquired DICOM images were then segmented by at least one experienced examiner using CilOCT, an open-source software implementing a semi-automated segmentation algorithm based on fitting polynomial splines to brightness gradients [10,32]. Subsequently, multiple parameters of the segmented images, the perpendicular axis (PA), the ciliary muscle area (CMA), the ciliary muscle thickness (CMT) profile, and the coordinates of the scleral spur (SP) and the ciliary muscle apex (CA), were automatically determined and exported [10,20]. All the settings used for the semi-automated segmentations are stored as an XML file, which allows for a reliable reproduction of the segmentation. For the full methodological protocols, we direct readers to the original articles. The studies referred to in this work followed the tenets of the Declaration of Helsinki and were approved by the Institutional Review Board of the Medical Faculty of the University of Tuebingen (376/2017BO2).

#### 2.1.2. Image Preparation

**Figure 1:**
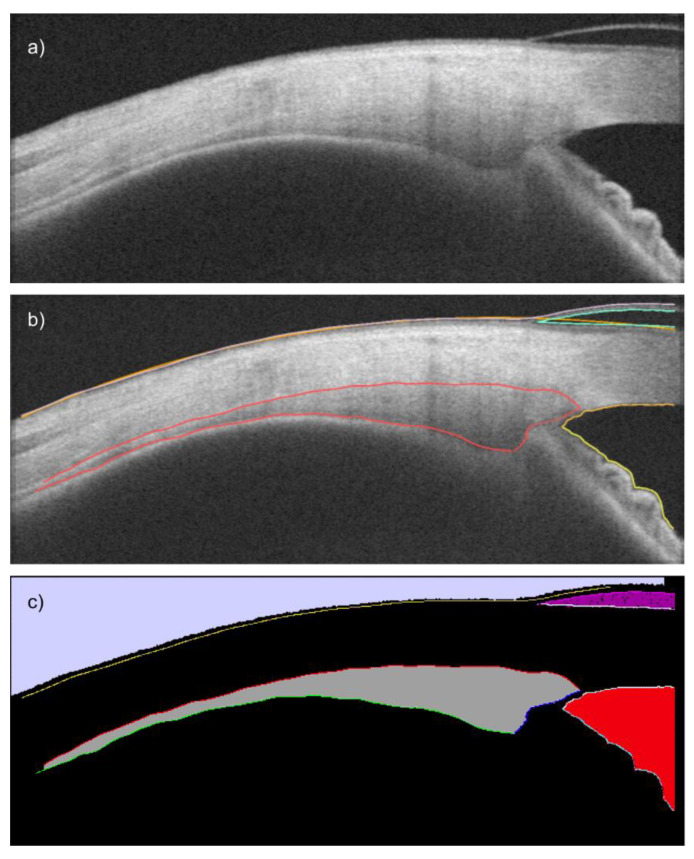
(**a**) OCT image of the ciliary muscle (here with eye wearing a soft contact lens) (feature image), (**b**) the semi-automated segmentation (red: ciliary muscle borders, orange: scleral-conjunctival boundary, turquoise: contact lens, yellow and orange: anterior chamber borders), (**c**) the label image based on image (**b**) with the 13 segmentation classes (Table 1) used as Ground truth for training the network.

The exported raw DICOM images of studies B and C were rotated and resized to 1280 × 512 pixels according to [22]. Subsequently, the images were segmented with built-in functions of the CilOCT software [32] using previously created XML segmentation files, and converted to feature (PNG, 640 × 480 pixels, 8 bit grayscale) and corresponding label images (PNG, 640 × 480 pixels, 8 bit palette RGB), representing the Ground truth with 13 segmentation classes (Table 1 and Figure 1). The downscaling is performed to allow a complete image to fit into the GPU memory. However, the convolutional network’s architecture does not employ a fully connected layer [34] and is therefore size-independent, i.e., the later inference using the trained network accepts original-sized images.

#### 2.1.3. Training and Validation Data

**Figure 2:**
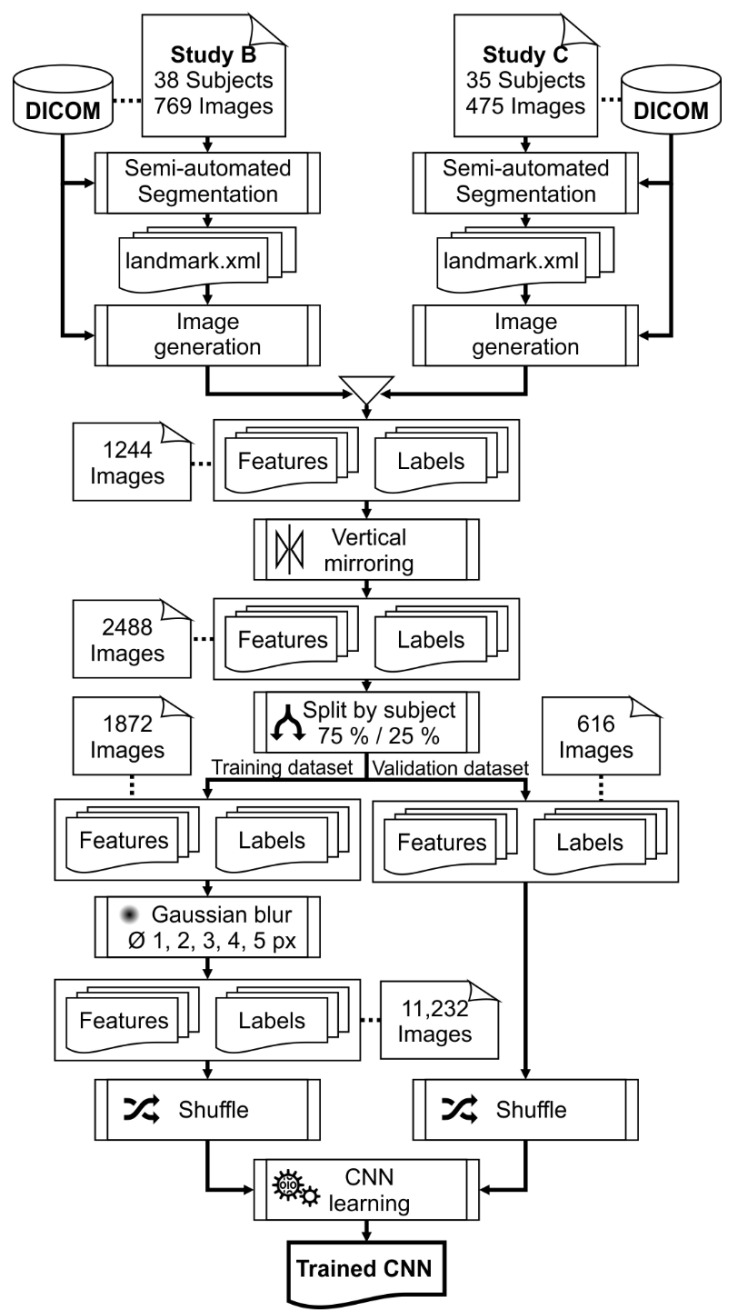
Workflow of the preparation of training and validation data. DICOM files are converted to feature and label (Ground truth) images using previously created segmentation files. All images are mirrored vertically, and the training data are additionally augmented using Gaussian blur of five different radii.

The OCT images (1244) of studies B and C were combined and split by subject into a training (75%) and a validation (25%) dataset, resulting in 936 images in the training dataset and 308 images in the validation dataset. The different recording conditions of the studies (the number of repeated measures, emmetropes vs. myopes, accommodative demand, near vs. far accommodation, and pre- vs. post-nearwork) were kept balanced between both datasets, whereby the images of one subject were either assigned to the training or the validation dataset. Subsequently, the images of both datasets were mirrored vertically to double the number of images available for training and validation, and to enable the network to learn the segmentation of the OCT images of the left eye’s ciliary muscle (training: 1872; validation: 616 images). The images of the training dataset were further augmented by blurring with ImageMagick [35] using five different radii (1–5 px) of a Gaussian blur. Blurring allows for the simulation of the poor image quality resulting from suboptimal recording conditions. After the data augmentation, 11,232 images for training and 616 images for validation were available. To prevent any bias or order of the images, the image files were shuffled by randomly renaming them using their SHA-1 hash. Figure 2 depicts the workflow for creating training and validation data.

### 2.2. Network Architecture

*Ciloctunet* uses the Freiburg U-Net network’s [33] architecture with some modifications: between the convolution and activation (ReLU) layer pairs and pooling layers, batch normalization layers were inserted to allow for a faster training and improved regularization and to avoid an overfitting [36] in favor of the dropout layers used in the original architecture. Furthermore, the classification comprises 13 different classes (Table 1), compared to two classes (foreground/background) in the U-Net. Since the frequencies of the pixels belonging to a certain class are highly unbalanced, e.g., they are much higher for the background pixels compared to the pixels representing the boundaries of the ciliary muscle, the SoftMax loss layer was replaced by an Infogain multinomial logistic loss layer. This allows for the individual weighting of the loss for each class, thus penalizing the misclassifications of the underrepresented classes. The Infogain loss is mathematically formulated as in Equation (1), where *E* is the loss, *N* is the number of images, *K* is the number of classes, *l_n_* is the class Ground truth of the *n*th sample classified to the *k*th class, and *p_n,k_* is the probability of the *n*th sample classified to the *k*th class, satisfying $\overset{K}{\underset{k}{\sum }}p_{n,k}=1$ and $p_{n,k}\geq 0$ [37].

(1) $$E=-\frac{1}{N}\overset{N}{\underset{n=1}{\sum }}\overset{K}{\underset{k=1}{\sum }}H_{l_{n},k}\left(p_{n},k\right)$$

$H_{l_{n},k}$ is the Infogain weight for the *n*th sample with the Ground truth *l_n_* to be classified to class *k* [37]. The Infogain matrix with the weights is calculated using a custom Python script for each image separately based on the relative proportions of the number of pixels belonging to the different classes within an image during the training phase.

### 2.3. Network Training

The network was implemented with the Caffe 1.0 deep learning framework [38]. The training was performed using the Nvidia Deep Learning GPU Training System (DIGITS) version 5 with Python 2.7 on an Ubuntu 18.04 LTS system with two GeForce GTX 1080 Ti (12 GB) graphics cards. The training leveraged the RMSprop optimizer with a decay value of 0.99 and a learning rate of 1e-05 and a batch size of two. The weights were initialized using the MSRA weight filler [39].

### 2.4. Testing

For testing, the 180 OCT images acquired in study A were used, comprised of six images per subject (session 1: images 1–3; session 2: images 4–6) for near and distance vision, respectively. The semi-automated segmentation of the two examiners was compared with those performed by the network, which had not “seen” the images before. Therefore, the CilOCT software was extended to use the trained network as an alternative to the semi-automated segmentation process for single images as well as for batch execution. The integration into the software is based on JavaCV (version 1.5.3), the Java bindings of OpenCV (version 4.3.0) [40], and the OpenCV Deep Neural Network (DNN) module.

**Figure 3:**
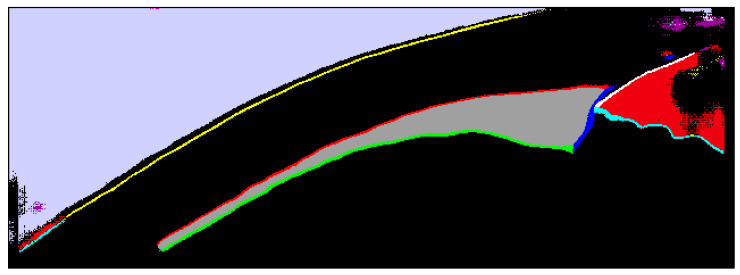
An exemplary result of the segmentation of an OCT image of the ciliary muscle performed by *Ciloctunet*. The colors correspond to the segmentation classes listed in Table 1. There are several wrong classifications, however, most of them only affect areas that are not used for further processing.

*Ciloctunet* outputs the segmentation results as a two-dimensional matrix of the pixels’ probabilities to belong to a particular segmentation class. Pixels with probabilities lower than five are discarded and those belonging to the segmentation classes representing the borders are skeletonized using a Java implementation of the Zhan–Suen thinning algorithm [41,42]. The remaining pixels are clustered using DBSCAN (ε = 30, minimum points = 20) [43] and the pixels which are not part of a cluster are removed. Both skeletonization and clustering help to discard possible spurious segmentation results (i.e., isolated wrongly classified pixels, Figure 3) and simplify the later fitting of the polynomial splines applied in the CilOCT software. The fitted splines not only allow for the segmentation of the ciliary muscle but are also used to determine the borders of the different types of tissues. These borders are subsequently used to correct for image distortion caused by the different refractive indices of the corresponding tissue.

### 2.5. Statistical Analysis

The accuracy of the segmentation was evaluated with the intersection-over-union (IoU) metric [44] between the ciliary muscle area (CMA, class 8, Table 1) resulting from the segmentation performed by the network and the two examiners (SW and TS). Furthermore, descriptive statistics of the differences between the Cartesian coordinates of the anatomical landmarks ciliary muscle apex and the scleral spur resulting from the particular segmentations were calculated. Both the IoU calculation and descriptive statistics were performed before the clustering and skeletonization.

Based on the segmentation, the biometric parameters PA and CMA were extracted after the distortion correction as described in [10]. Two linear mixed-effects models with the fixed effects segmenter (CNN, SW, and TS), session (1, 2), accommodative state (far and near) and their interactions, and the participant as a random effect were fit by the restricted maximum likelihood estimation (REML) to assess the significance of the effects in explaining the variations of the dependent variables PA and CMA, respectively. The variance inflation factors (VIF) of the predictors were calculated and assured to fall well below the common threshold value, indicating no collinearity between them [45]. The residuals were confirmed visually to follow a normal distribution and the homogeneity of the variances was ensured using the Brown–Forsythe test [46,47].

Paired-sample *t*-tests were conducted to compare the biometric parameters PA and CMA derived from the segmentations performed by the two examiners and the network, and the limits of agreement (LoA) were calculated according to the Bland–Altman method [48]. Additionally, two-way mixed intra-class correlation coefficients (ICC) with an average measure and absolute agreement between the segmentations were calculated [49].

The similarity of the CMT profiles between the manual and network segmentation was evaluated by calculating a modified IoU metric according to (Equation (2)) as the ratio of the summed minimum and maximum CMT values up to a −4.5 mm distance from the scleral spur of the two segmentations (*Ciloctunet* vs. SW, *Ciloctunet* vs. TS, SW vs. TS) of a particular OCT image. The IoU results of the single OCT images were then averaged. In the case of a perfect alignment, the IoU would be 1.0 (Equation (2)).

(2) $${\mathrm{CMT}\;\mathrm{IoU}}_{seg1/seg2}=\frac{\sum_{x}min\left({\mathrm{CMT}}_{seg1}\left(x\right),{\;\mathrm{CMT}}_{seg2}\left(x\right)\right)}{\sum_{x}max\left({\mathrm{CMT}}_{seg1}\left(x\right),{\mathrm{CMT}}_{seg2}\left(x\right)\right)}$$

Furthermore, the mean and standard deviations of the mean absolute error (MAE) [50] (in pixel) between the CMT profiles derived from the different segmentations were calculated.

To test the applicability of the segmentation performed by *Ciloctunet*, paired-sample *t*-tests were conducted to compare the averaged biometric parameters PA and CMA between the near and far accommodation. The results were contrasted with those reported by Wagner et al. [10].

All statistical analyses were performed using SAS JMP 15.1 (SAS Institute Inc., Cary, NC, USA). The ICCs were calculated using IBM SPSS Statistics 26.0 (IBM Corp., Armok, NY, USA).

## 3. Results

### 3.1. Training Performance

The training was stopped after 30 epochs and about 18.5 h. At that time, an accuracy of 97.5% for the validation dataset was reached. During the training, after about 11 epochs (validation accuracy of 96.4%), a slight increasing of the validation loss could be observed, whereas the training loss continued to decrease.

### 3.2. Segmentation Accuracy

Figure 3 exemplifies the result of the segmentation of an OCT image of the ciliary muscle performed by *Ciloctunet*. The colors correspond to the segmentation classes as listed in Table 1. The image contains several spurious and wrong segmentations. However, most of them are related to areas not used for further processing, which only requires the borders of the ciliary muscle (red, green, and blue), as well as the borders between different tissues or between air and tissue (yellow, white, and cyan). Most wrong classifications of these borders are removed by skeletonization and clustering.

Dataset A, used for testing the trained network, comprises 180 images, whereby some images were discarded from segmentation by the examiners due to a bad image quality (SW: 3; TS: 1). The segmentations performed by Ciloctunet and the first examiner (SW) showed a median IoU of 0.84 (Q25: 0.78, Q75: 0.87; *n* = 177), comparable to the median IoU of 0.84 (Q25: 0.81, Q75: 0.86; *n* = 176) between segmentations of the first (SW) and the second (TS) examiner.

The median IoU between the segmentations of the second examiner (TS) and *Ciloctunet* was 0.80 (Q25: 0.77, Q75: 0.83; *n* = 179).

### 3.3. Ciliary Muscle Apex and Scleral Spur Coordinates

**Figure 4:**
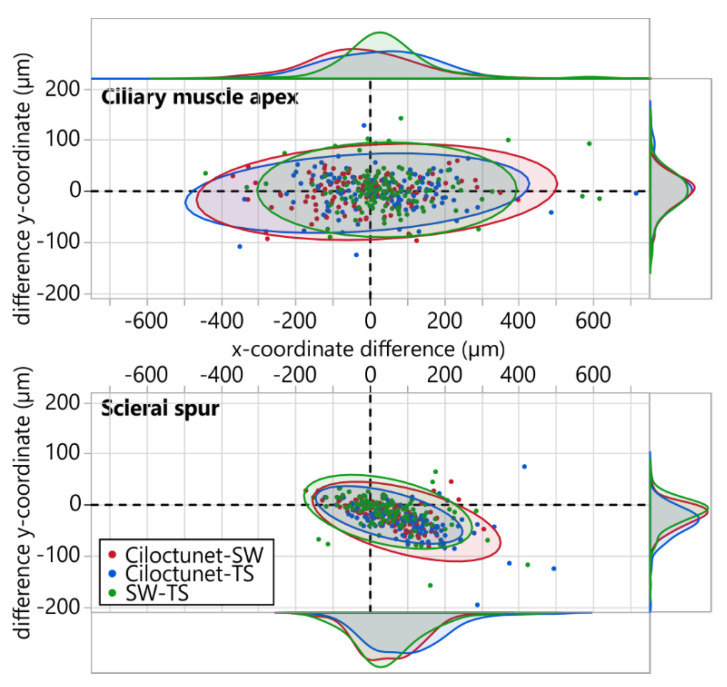
Distribution of the differences of the *x*/*y*-coordinates of the ciliary muscle apex and the scleral spur between different pairs of segmentation and the corresponding 95% density ellipses (red: *Ciloctunet* vs. SW; blue: *Ciloctunet* vs. TS; green: SW vs. TS). The density plots depict the shape of the distribution of the differences between the *x*- and *y*-coordinate of the respective pairs of segmentations.

The distributions of the differences between the *x*/*y*-coordinates of the anatomical landmarks ciliary muscle apex and scleral spur derived from the different segmenters are visualized in Figure 4. The results of the descriptive statistics of the mean and absolute differences of the *x*- and *y*-coordinates, as well as of the Euclidean distances, are given in Table 2.

### 3.4. Effect of Segmenter on Biometric Parameters

Both models’ residuals follow a normal distribution and their variances are homoskedastic. The linear mixed-effects model with the dependent variable PA (*n* = 380, R^2^_adj._ = 0.73) revealed a statistically significant effect for the accommodative demand (distance), but not for the segmenter, the session, or their interactions (Table 3). The linear mixed-effects model with the dependent variable CMA (*n* = 380, R^2^_adj._ = 0.54) revealed a statistically significant effect for the accommodative demand (distance) and the segmenter, but not for the session, or any interactions (Table 3).

**Figure 5:**
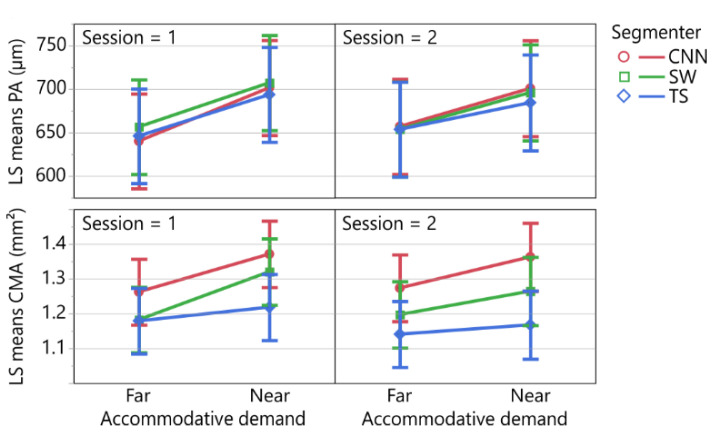
Least-square means plots of the results of the linear mixed-effects models for the dependent variables perpendicular axis (PA, **top**) and ciliary muscle area (CMA, **bottom**) for the fixed effects accommodative demand, segmenter, and session. In both models, the accommodative demand was found to be statistically significant. Only for the CMA, the segmenter was shown to also have a statistically significant effect.

A post hoc comparison using a *t*-test indicated a statistically significant mean difference of 53.25 (95% CI: [38.70, 67.80]) µm between the least-square means (±SE) of the far (647.58 ± 24.51 µm) and near (700.83 ± 24.57 µm) conditions (*t* (356.0616) = 7.198, *p* < 0.0001). The top of Figure 5 depicts the differences in the least-square means of the PA with the effects segmenter, accommodative demand (distance), and session.

A post hoc comparison using a *t*-test indicated a statistically significant mean difference of 0.0953 (95% CI: [0.0557, 0.1350]) mm^2^ between the least-square means of the far (1.2080 ± 0.0410 mm^2^) and near (1.3033 ± 0.0413 mm^2^) conditions (*t* (356.1653) = 4.7269, *p* < 0.0001).

A Tukey HSD post hoc test revealed statistically significant differences (*p* < 0.0001) of 0.0654 (95% CI: [0.0072, 0.1236]) mm^2^ between the least-square means of the segmentation performed by the neural network (mean ± SE: 1.3168 ± 0.0424 mm^2^) and examiner SW (mean ± SE: 1.2514 ± 0.0423 mm^2^), and of 0.1181 (95% CI: [0.0601, 0.1761]) mm^2^ (*p* = 0.0232) between the neural network and examiner TS (1.987 ± 0.0423 mm^2^, but not between the examiners SW and TS (*p* = 0.0807). The bottom of Figure 5 shows the least-squares means differences in the CMA concerning the interaction of the accommodative demand, segmenter, and session.

### 3.5. Repeatability Analysis of the Biometric Parameters

Paired samples *t*-tests between the biometric parameters PA and CMA derived from the segmentation performed by *Ciloctunet* and the examiners SW and TS revealed statistically significant differences only for the CMA but not for the PA (Table 4).

The CMA calculated from the segmentation by *Ciloctunet* differed from those of SW and TS by −0.08 ± 0.19 mm^2^ and −0.14 ± 0.19 mm^2^, respectively. No statistically significant difference was found between the biometric parameters derived from the *Ciloctunet* segmentation of the first (OCT images 1–3) and the second session (images 4–6).

The inter-rater reliability between *Ciloctunet* and the two examiners was good (with outliers) to excellent (without outliers) for the PA, and moderate (with outliers) to good (without outliers) for the CMA, according to the classification of the ICC of [49]. The intra-rater reliability between the first and the second session segmented by *Ciloctunet* was moderate (with outliers) to excellent (without outliers) for the PA and good for the CMA (Table 4).

### 3.6. Comparison of Ciliary Muscle Thickness Profiles

**Figure 6:**
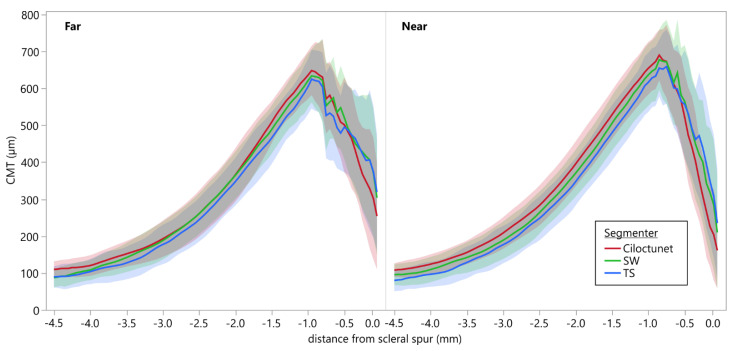
Averaged CMT profiles of dataset A during near and far accommodation derived from the different segmenters SW (green), TS (blue), and *Ciloctunet* (red). The shaded areas denote one standard deviation.

Figure 6 depicts the averaged CMT profiles with standard deviations derived from the segmentation of the OCT images of dataset A performed by the two examiners SW and TS, as well as by *Ciloctunet*. It is evident that the CMT profiles resulting from the segmentation performed by *Ciloctunet* are slightly thicker; the ones of TS are slightly thinner than the ones of SW. The average MAE (±SD) between *Ciloctunet* and SW is 35.16 ± 12.84 µm, between *Ciloctunet* and TS 45.86 ± 17.97 µm, and between SW and TS 34.99 ± 15.71 µm. Accordingly, the mean (±SD) IoU between *Ciloctunet* and SW (0.89 ± 0.04) is higher than between *Ciloctunet* and TS (0.86 ± 0.05) or between SW and TS (0.89 ± 0.05).

### 3.7. Replication of the Results of Study C by Comparison of Biometric Parameters Derived from Ciloctunet Segmentations during Near and Far Accommodation

Results for the parameters PA and CMA of up to six OCT images per subject (*n* = 13) and condition (far and near) were averaged after a ciliary muscle segmentation with *Ciloctunet*. The paired-samples *t*-tests revealed statistically significant differences for the averaged PA (−52.64 ± 39.98 µm, *t* (12) = 4.7472, *p* = 0.0005) and for the CMA (−0.10 ± 0.06 mm^2^, *t* (12) = 6.1804, *p* < 0.0001) during near (PA: 702.58 ± 87.51 µm; CMA: 1.36 ± 0.17) and far accommodation (PA: 649.94 ± 59.91 µm; CMA: 1.27 ± 0.16 mm^2^).

## 4. Discussion

The deep neural network *Ciloctunet*, which leverages the Freiburg U-Net convolutional network architecture [33], was trained to perform an automated segmentation of the ciliary muscle in the AS-OCT images using data from two previously published studies [12,20]. The Freiburg U-Net architecture was chosen since it aims to lower the number of required samples to train the network by using annotated data more efficiently. In contrast to most other application areas of the Freiburg U-Net, which focus on the segmentation of areas, the derivation of the ciliary muscle’s biometric parameters and the prior distortion correction requires the segmentation of the muscle’s borders (Figure 1). Therefore, the SoftMax loss layer of the net was replaced by an Infogain loss layer, which weights the loss according to the ratio of pixels belonging to the different segmentation classes, thus addressing the problem of a class imbalance, that could otherwise result in a high accuracy simply by classifying everything as the background. Furthermore, the Infogain loss has been shown to achieve a better performance than the cross-entropy loss [37]. Using other loss functions like the Dice coefficient, which works similarly to the IoU metric and allows for dealing with the class imbalances [51], a focal loss, as suggested by [52], or a combination of both [53], could further improve the performance and will be evaluated in the future. An alternative approach could use network architectures tailored to edge or contour detection like the holistically nested edge detection (HED) network [54,55]. However, in this study, the Freiburg U-Net led to better results than HED Future work might evaluate different network architectures, like the DeconvNet, SegNet, DeepLabv3+, Criss-Cross Network (CCNet), or Context Encoding Network (EncNet), for further improving the accuracy of the segmentation. Cabeza-Gil et al. recently published a comparison of several CNN architectures (U-Net and LinkNet, both with different backbone structures, like MobileNetv2, Vgg19, and EfficientNetb4) for the segmentation of the ciliary muscle in OCT images [53] and found the U-Net to have the highest performance compared to the others [53].

*Ciloctunet* was trained for 30 epochs, though after 11 epochs the validation loss stopped decreasing, indicating that the network started to overfit the data. However, since the increase in the loss was minor, we decided against an early stopping [56]. Overfitting could be avoided by increasing the number of the training images, for instance by including the segmentation data of other examiners. This would probably also improve the generalization and increase the accuracy, which could also be achieved alternatively by a further augmentation of the training dataset. Currently, the OCT images are augmented by mirroring, which not only increases the generalization but also allows for the segmentation of images taken from the left eye, and by Gaussian blurring with different kernel sizes, which simulates low OCT image qualities. Additionally, the warping of the images using elastic deformation [57,58] or modifying the image contrast either globally or locally could be applied. Other methods could also be beneficial [59].

*Ciloctunet* leverages 13 different segmentation classes (Table 1), whereby only a subset of them representing the tissue borders is used for the subsequent processing (distortion correction, polynomial spline fit, and the calculation of the biometric parameters). The additional segmentation classes were provided as an aid for the training of the network, since the Infogain loss penalizes overlapping areas.

The comparison of the segmentation results based on the parameter ciliary muscle area (class 8, Table 1) showed a good to very good IoU of 0.84, similar to the IoU of 0.84 between the examiner SW and examiner TS. The lower IoU of 0.80 between the segmentations of *Ciloctunet* and TS indicates that *Ciloctunet* probably resembles the characteristics of examiner SW, who performed the segmentation of the datasets B and C, which were used as Ground truth for the training. When evaluating this outcome, one has to take into consideration that the IoU metric is calculated without the removal of spurious segmentation results (Figure 3) using skeletonization and clustering, which is performed before a further analysis.

Two important anatomical landmarks of the ciliary muscle, the scleral spur and the ciliary muscle apex, were analyzed separately by comparing the differences in the absolute coordinates between the pairs of segmentations of the two examiners and *Ciloctunet* (Table 2). Interestingly, the variability of the differences is higher along the *x*-axis than along the *y*-axis for both the scleral spur and the ciliary muscle apex, whereby the variability of the differences for the scleral spur is, in general, smaller (Figure 4). The median Euclidean distance between the scleral spur coordinates derived from the segmentation of *Ciloctunet* and examiner SW is 67.44 µm with an interquartile range (IQR) of 87.65 µm. This corresponds to the values between the two examiners SW and TS, with a slightly lower median Euclidean distance of 60.94 µm and IQR of 83.40 µm. The median Euclidean distance between *Ciloctunet* and examiner TS is 99.92 (IQR: 127.09) µm. A previous study investigating the variability of the ciliary muscle’s segmentation in the OCT images of six subjects [60] reported an average inter-examiner difference in the scleral spur coordinates (presumably the Euclidean distance) of 122 µm and an intra-examiner standard deviation of 29 µm. Assuming a normal distribution, this corresponds to an IQR of 39.12 µm (=2 * 0.6745 * SD) [61,62], whereby the coordinates of the scleral spur were averaged over 10 images per subject. Ref. [63] trained a convolutional neural network to mark the position of the scleral spur in the AS-OCT images of 921 eyes and reported a CNN prediction error of the absolute coordinates (Euclidean distance) compared to the results of an experienced examiner of 73.08 µm with a standard deviation of 52.06 µm, which corresponds to an IQR of 70.23 µm, assuming a normal distribution. The reported inter-grader difference was 97.34 µm with a standard deviation of 73.29 (IQR: 98.87) µm.

The evaluation of the possible effects of the segmenter, distance, and session on the biometric parameters PA and CMA using linear mixed-effect models revealed an expected statistically significant effect of the accommodative demand for both parameters. A statistically significant effect of the segmenter was only present for the CMA, but not for the PA. These results indicate that for the PA, the segmenter is interchangeable, whereas for the CMA, *Ciloctunet* constantly overestimates and the examiner TS constantly underestimates the area of the ciliary muscle compared to examiner SW (Figure 5). Nevertheless, both segmenters as well as *Ciloctunet* detected the difference in the CMA between near and far accommodation. The comparison of the morphological changes in the ciliary muscle during near and far accommodation based on the segmentation of *Ciloctunet* resulted in statistically significant differences in both the PA and CMA (Figure 5), in the same range as those reported by [10]. The PA increased by about 53 µm from the far to near condition (Wagner et al.: ~43 µm), the CMA by about 0.1 mm^2^ (Wagner et al.: ~0.1 mm^2^).

The mean difference between the PA derived from the segmentation of *Ciloctunet* and the two examiners was 5.35 µm (*Ciloctunet*–SW) and −3.80 µm (*Ciloctunet*–TS), respectively, which is smaller than the mean difference of −9.60 µm between the two examiners (SW–TS) and considerably lower than those reported by [60] for the comparable parameter CMTMAX, derived from segmentation of two examiners (relaxed ciliary muscle: 20 µm, accommodated ciliary muscle: 25 µm). Cabeza-Gil et al. report a mean difference of 1.2 µm with a standard deviation of about 23.72 µm between CMTMAX derived from CNN-based segmentations and those performed by a human expert [53], therefore slightly better than the difference between CNN and human examiners found in this study.

The variability expressed as the standard deviation of the parameter PA between the first and second session of the segmentations performed by *Ciloctunet* is 84.07 µm, thus about the same as that reported by [60] (54 to 77 µm), taking into consideration that the standard deviation decreases with the square root of the number of samples (*n* = 6 subjects × 10 images). Table 5 summarizes the inter-examiner as well as the CNN-examiner differences as reported by several studies.

The average mean absolute error (MAE) of the CMT profiles between the segmentations of *Ciloctunet* and examiner SW is 35.16 µm and between *Ciloctunet* and examiner TS is 45.86 µm. Both are in the same range as the averaged MAE of 34.99 µm between the two examiners and about 2–3 times the axial resolution of 18 µm of the Zeiss Visante AS-OCT. Converted to pixels, this corresponds to a difference of approximately 4–5 pixels. Accordingly, the comparison of the CMT profiles shows high IoU values, indicating a high agreement of the CMT profiles derived from the different segmenters.

Interestingly, a statistically significant difference between the segmentations of *Ciloctunet* and the examiners was found only for the CMA and not for the PA (Table 4). This is probably explained by the summation of slight differences in the segmentation of the muscles’ boundaries along the extent of the ciliary muscle. Dividing the mean CMA difference of 0.08 mm^2^ between *Ciloctunet* and examiner SW by the length of 4 mm (taken from the scleral spur) used for calculating the CMA [10] results in an approximate difference of 20 µm or about 2.6 px per mm. This corresponds to a slight increase of two pixels in the ciliary muscle thickness (distance between the upper and lower boundaries). Therefore, although statistically significant, the difference is not clinically relevant. The summation of the differences seems to render selective thickness measurements, like the CMTMAX and perpendicular axis, or continuous ciliary muscle thickness profiles to be favorable over the ciliary muscle area for comparisons, given that the segmentations are not performed by only a single examiner. The application of *Ciloctunet*, currently trained using the segmentation of a single examiner as the Ground truth, avoids these differences. Furthermore, it also avoids a possible training effect in segmenting over time, which was observed by [10].

The analysis of the biometric parameters only uses some segmentation classes for the optical distortion correction, namely the boundaries of the ciliary muscle, the air-scleral border, and the borders to the anterior segment. While the definition of the ciliary muscle boundaries does not require each segmentation class (Table 1), they are needed for the distortion correction caused by the refractive indices of the different tissues. Furthermore, they could be used for other applications like the measurement of the scleral thickness [64], the scleral curvature [65], the segmentation of the angle recess and the trabecular iris space area, or the determination of the iridocorneal angle [1], which is used for the automatic detection of the angle closure [66,67].

## 5. Conclusions

By leveraging existing datasets from previous studies for training, validation, and testing, *Ciloctunet* not only proved the feasibility of the automated segmentation of the ciliary muscle in AS-OCT images like a similar approach published recently [53], but moreover demonstrated to be on par with experienced examiners. Thereby, *Ciloctunet* enables the analysis of high numbers of images of large study cohorts by avoiding a time-consuming manual segmentation with possible examiner biases. To the best of our knowledge, *Ciloctunet* is the first open-source solution for the fully automated segmentation of the ciliary muscle in AS-OCT images, which, since integrated into the open-source software CilOCT, leverages well-established workflows. *Ciloctunet* is available for download at https://github.com/strator1/Ciloctunet, accessed on 1 October 2022.

## Figures and Tables

**Table 1:** Segmentation classes and their corresponding colors, as used in the labeled images (Ground truth).

#	Segmentation Class	Color	RGB Value
1	Background	● Black	0, 0, 0
2	Inner scleral border	○ White	255, 255, 255
3	Upper iris border	● Cyan	0, 255, 255
4	Outer conjunctiva border	● Yellow	255, 255, 0
5	Outer ciliary muscle border	● Red	255, 0, 0
6	Inner ciliary muscle border	● Green	0, 255, 0
7	Vertical ciliary muscle border	● Blue	0, 0, 255
8	Ciliary muscle area	● Dark gray	160, 160, 160
9	Upper contact lens border ^1^	● Magenta	240, 0, 240
10	Lower contact lens border ^1^	● Light gray	240, 240, 240
11	Contact lens area ^1^	● Purple	160, 0, 160
12	Anterior chamber area	● Sienna	240, 0, 15
13	Air area	● Lavender	209, 209, 255

^1^ Refers to an optionally present soft contact lens.

**Table 2:** Descriptive statistics of the differences of the respective *x*/*y*-coordinates of the ciliary muscle apex and the scleral spur between different pairs of segmentations.

	*Ciloctunet*–SW	*Ciloctunet*–TS	TS–SW
Ciliary muscle apex (*n* = 130)			
Difference *x* Mean ± SD (µm)	−21.82 ± 139.27	30.89 ± 148.86	46.15 ± 142.43
Difference *y* Mean ± SD (µm)	−3.66 ± 31.08	−1.03 ± 37.26	2.27 ± 38.00
Absolute difference *x* Median [Q25, Q75] (µm)	85.16 [44.53, 147.66]	101.17 [55.66, 153.71]	60.16 [24.61, 110.94]
Absolute difference *y* Median [Q25, Q75] (µm)	18.75 [9.37, 33.40]	21.68 [11.72, 36,91]	20.51 [8.79, 38.38]
Euclidean distance Median [Q25, Q75] (µm)	89.38 [54.72, 152.11]	111.17 [65.69, 156.24]	71.44 [34.62, 127.54]
Scleral spur (*n* = 131)			
Difference *x* Mean ± SD (µm)	51.68 ± 80.61	98.63 ± 103.32	48.99 ± 92.32
Difference *y* Mean ± SD (µm)	−20.16 ± 23.10	−33.13 ± 31.87	−14.11 ± 29.62
Absolute difference *x* Median [Q25, Q75] (µm)	65.23 [27.34, 114.06]	94.53 [35.16, 157.03]	58.59 [25.39, 112.50]
Absolute difference *y* Median [Q25, Q75] (µm)	19.92 [8.20, 36.62]	31.05 [15.82, 48.05]	15.23 [4.98, 30.47]
Euclidean distance Median [Q25, Q75] (µm)	67.44 [29.61, 117.26]	99.92 [45.94, 173.03]	60.94 [31.83, 115.23]

**Table 3:** Results of the linear mixed-effects models with the dependent variables perpendicular axis (PA) and ciliary muscle area (CMA).

Fixed Effect	PA (*n* = 380, R^2^_adj._ = 0.73)		CMA (*n* = 380, R^2^_adj._ = 0.54)	
	*F*-Statistic	*p*-Value	*F*-Statistic	*p*-Value
Distance	*F* (1, 356.17) = 71.2319	<0.0001	*F* (1, 356.47) = 27.6147	<0.0001
Segmenter	*F* (2, 356.00) = 0.9343	0.3938	*F* (2, 355.99) = 30.6360	<0.0001
Session	*F* (1, 356.28) = 0.0003	0.9852	*F* (1, 356.77) = 2.0181	0.1563
Distance x Segmenter	*F* (2, 356.00) = 0.5168	0.5969	*F* (2, 356.00) = 2.3787	0.0941
Distance x Session	*F* (1, 356.27) = 1.6976	0.1934	*F* (1, 356.75) = 1.3051	0.2541
Segmenter x Session	*F* (2, 356.00) = 0.6444	0.5256	*F* (2, 355.99) = 0.8143	0.4438
Distance x Segmenter x Session	*F* (2, 356.00) = 0.0712	0.9313	*F* (2, 356.01) = 0.3828	0.6823

**Table 4:** Results of the paired samples *t*-tests between the biometric parameters perpendicular axis (PA) and ciliary muscle area (CMA), derived from the segmentations performed by the two examiners SW and TS, and *Ciloctunet*.

Comparison	Mean Diff. ± SD	*t*-Statistic ^a^			Limits of Agreement	ICC ^b,d^ [95% CI]	ICC ^b,c,d^ [95% CI]
		df	*t*-Value	*p*-Value			
Perpendicular axis (µm)							
*Ciloctunet*–SW	5.35 ± 67.64	119	0.8668	0.3878	[−127.21, 137.92]	0.85 [0.79, 0.90]	0.93 [0.90, 0.95]
*Ciloctunet*–TS	−3.80 ± 77.10	122	−0.5473	0.0585	[−154.93, 147.32]	0.82 [0.74, 0.87]	0.90 [0.86, 0.93]
SW–TS	−9.60 ± 57.11	124	−1.8793	0.0626	[−121.54, 102.34]	0.91 [0.88, 0.94]	0.91 [0.88, 0.94]
*Ciloctunet*, Session 1–2	13.23 ± 84.07	49	1.1132	0.2711	[−151.54, 178.01]	0.70 [0.47, 0.83]	0.93 [0.87, 0.96]
Ciliary muscle area (mm²)							
*Ciloctunet*–SW	−0.08 ± 0.19	119	−4.8045	<0.0001	[−0.45, 0.29]	0.65 [0.45, 0.77]	0.71 [0.44, 0.83]
*Ciloctunet*–TS	−0.14 ± 0.19	122	−7.8459	<0.0001	[−0.51, 0.24]	0.58 [0.19, 0.76]	0.58 [0.19, 0.76]
SW–TS	−0.06 ± 0.21	124	−3.2455	0.0015	[−0.47, 0.35]	0.63 [0.47, 0.74]	0.67 [0.53, 0.77]
*Ciloctunet*, Session 1–2	0.00 ± 0.17	49	−0.1058	0.9162	[−0.34, 0.33]	0.75 [0.57, 0.86]	0.75 [0.57, 0.86]

^a^ paired-samples *t*-test, all differences were normally distributed, ^b^ ICC: two-way mixed, average measure, absolute agreement, ^c^ ICC with two outliers excluded, ^d^ ICC classification: <0.5: poor; <0.75: moderate; <0.9: good; ≥0.9: excellent [49].

**Table 5:** Overview of inter-examiner and CNN-examiner differences between absolute coordinates of the scleral spur and the biometric parameter ciliary muscle thickness (CMTMAX, PA) derived from segmented OCT images as reported by different studies.

	Euclidean Distance between Absolute Scleral Spur Coordinates (µm)							
	Inter-Examiner Difference				CNN-Examiner Difference			
	Mean/Median		SD	IQR	Mean/Median		SD	IQR
Current study	60.94			83.40	SW: TS:	67.44 99.92		87.65 83.40
Chang et al. (2018) [60]	122							
Xu et al. (2020) [63]	97.34		73.29	98.87 ^1^	73.08		52.06	70.23 ^1^
	Pointwise ciliary muscle thickness difference: CMTMAX/PA mean (µm)							
	Mean		SD		Mean		SD	
Current study	−9.60		57.11		SW: TS:	5.35 −3.80	67.64 77.10	
Chang et al. (2018) [60]	Relaxed: Accommodated:	20 25	69.57 ^2,3^ 34.79 ^2,3^					
Cabeza-Gil et al. (2022) [53]					1.2		23.72 ^4^	

^1^ IQR calculated from the standard deviation according to [61,62], assuming a normal distribution: IQR = 2 * 0.6745 * standard deviation); ^2^ corrected for averaging of 10 images by a factor of square root of 10; ^3^ estimated from Bland–Altman plot; ^4^ calculated from Bland–Altman Limits of Agreement.

## Data Availability

The Ciloctunet model structure, as well as the trained model, are available at https://github.com/strator1/Ciloctunet under an open-source license (GPLv3). For easy application, Ciloctunet was integrated into CilOCT, a software for the semi-automated segmentation and analysis of the ciliary muscle in OCT images, available at https://github.com/strator1/CilOCT, accessed on 1 October 2022.
